# Ictal phase-amplitude coupling as a biomarker for seizure onset zone in neocortical epilepsy

**DOI:** 10.3389/fneur.2025.1632484

**Published:** 2025-09-03

**Authors:** Kazuki Nishioka, Yasushi Iimura, Takumi Mitsuhashi, Hiroharu Suzuki, Tetsuya Ueda, Kazuki Nomura, Yao Miao, Toshihisa Tanaka, Hiroshi Otsubo, Hidenori Sugano, Akihide Kondo

**Affiliations:** ^1^Department of Neurosurgery, Juntendo University, Tokyo, Japan; ^2^Epilepsy Center, Juntendo University Hospital, Tokyo, Japan; ^3^Shenzhen Technology University, Shenzhen, China; ^4^Department of Electrical Engineering and Computer Science, Tokyo University of Agriculture and Technology, Tokyo, Japan; ^5^Division of Neurology, The Hospital for Sick Children, Toronto, ON, Canada; ^6^Sugano Neurosurgery Clinic, Tokyo, Japan

**Keywords:** high-frequency oscillations, modulation index, phase-amplitude coupling, neocortical epilepsy, ictal EEG analysis

## Abstract

**Objective:**

To assess ictal phase–amplitude coupling (PAC) dynamics during seizure generation and explore whether ictal PAC analysis can help identify the seizure onset zone (SOZ) in neocortical epilepsy.

**Methods:**

We analyzed ictal intracranial electroencephalography recordings from 22 seizures in six patients with drug-resistant neocortical epilepsy who achieved International League Against Epilepsy Class I outcomes following resection surgery. PAC strength was quantified using the modulation index (MI), computed by coupling the amplitude of high-frequency bands (ripples: 80–200 Hz; fast ripples: 200–300 Hz) with the phase of slow-wave bands (1–2 Hz, 2–3 Hz, 3–4 Hz and 4–8 Hz). MI values were compared across SOZ, peri-SOZ (adjacent electrodes), and non-SOZ regions. Localization accuracy was evaluated using the area under the curve (AUC) from receiver operating characteristic curve analysis.

**Results:**

Across all PAC between ripples and slow wave bands, MI values were significantly higher in the SOZ compared to the peri-SOZ and non-SOZ regions (*p* < 0.001). During the late phase of seizure onset, MI_Ripples/4–8 Hz_ was also significantly higher in the peri-SOZ region than in the non-SOZ region (*p* < 0.001). MI_Ripples/4–8 Hz_ (AUC: 0.853) and MI_Ripples/3–4 Hz_ (AUC: 0.846) demonstrated strong SOZ localization performance. MI_Ripples/3–4 Hz_ distinguished SOZ from peri-SOZ (AUC: 0.800).

**Conclusion:**

Elevated MI during seizure onset accurately localized the SOZ in neocortical epilepsy. Ictal PAC may be a useful biomarker for identifying SOZ in neocortical epilepsy.

## 1 Introduction

The primary goal of epilepsy surgery is to remove the epileptogenic zone (EZ), identified based on clinical, electrophysiological, and imaging findings, to achieve seizure freedom (1). Identification of the EZ relies on clinical, electrophysiological, and imaging findings. However, because neocortical epilepsy can originate from any cortical region of the cerebral cortex, accurate localization of the EZ remains a major challenge. Favorable postoperative seizure outcomes have been reported when ictal electroencephalography (EEG) patterns are well localized or when lesions are completely resected (2, 3). While the seizure outcome is generally poor in patients with epileptogenic multi-foci or non-lesional epilepsy, resection of the seizure onset zone (SOZ) may still result in good outcomes (4, 5). However, unfavorable outcomes can occur when EZ overlaps with or lies near the eloquent cortex. In such cases, precise localization is crucial for determining appropriate resection margins and improving seizure outcomes.

When the epileptic focus cannot be identified by non-invasive evaluations, intracranial electroencephalography (IEEG) is indicated and performed (6, 7). Visual inspection of IEEG is time-consuming and requires expert interpretation. This limitation has prompted growing interest in quantitative and automated methods to enhance the accuracy of EZ localization.

Several quantitative biomarkers have been proposed, especially for interictal IEEG analysis, such as interictal epileptiform discharges (IEDs), high-frequency oscillations (HFOs), and entropy-based measures (8–10). Phase-amplitude coupling (PAC), a synchronous phenomenon between HFOs and slow waves (11), has been proposed as a quantitative marker of epileptogenicity (12–14). However, despite the development of these approaches, seizure outcomes after epileptic-focus resection have shown only modest improvement (15). As the SOZ and irritative zone often differ in anatomical location and spatial extent (1, 16), interictal analysis alone may be inadequate for precise localization of the EZ. Therefore, ictal EEG analysis is considered essential for accurately identifying the SOZ and delineating the EZ.

Recently, studies have suggested that ictal PAC may be a promising biomarker of epileptogenicity (17–20). Zhang et al. demonstrated that PAC between delta/theta/alpha phase and gamma/ripple amplitude increases significantly during the mid-seizure period in patients with temporal lobe epilepsy (21). Ueda et al. (22) used a sliding window technique to analyze ictal PAC in temporal lobe epilepsy and observed dynamic changes in the EZ during seizures. PAC between ripples and 3–4 Hz waves increased at seizure onset, briefly decreased, and then rose, whereas PAC between ripples and 4–8 Hz waves remained elevated throughout. Similarly, Hashimoto et al. reported that ripple–theta PAC is associated with seizure evolution, whereas ripple–delta PAC may contribute to seizure termination (19). In contrast, Xu et al. analyzed peri-ictal PAC dynamics and found that PAC decreased immediately after seizure onset, with different temporal trajectories observed between the seizure onset zone and propagation regions (23). These observations suggest that analyzing ictal PAC may help elucidate the mechanisms involved in seizure generation and termination. However, consensus is still lacking on key methodological issues such as analysis approaches, window selection, or the optimal combination of HFOs and slow-wave frequencies. Moreover, the functional roles of different PAC types during seizures remain unclear.

In neocortical epilepsy, rapid seizure propagation has been identified as a poor prognostic factor (3). Although a few studies have reported ictal PAC analysis in neocortical epilepsy (17, 18, 24), none have examined the spatiotemporal dynamics of PAC during the seizure onset phase, particularly through comparative analysis between the SOZ and surrounding areas. In the present study, we investigated ictal PAC using a sliding window technique between the SOZ and surrounding regions in patients with neocortical epilepsy, with a particular focus on seizure onset phase. We hypothesized that ictal PAC dynamics differ between the SOZ and surrounding regions during the seizure onset phase and that ictal PAC analysis can help localize the SOZ and distinguish it from surrounding regions. To test these hypotheses, we analyzed 30 s segments of ictal IEEG centered on seizure onset, computed PAC strength across regions, and evaluated whether ictal PAC analysis could accurately identify the SOZ.

## 2 Materials and methods

### 2.1 Patients

We reviewed 116 IEEG cases recorded between 2014 and 2021 at the Juntendo University Epilepsy Center (Tokyo, Japan). Subdural grid and depth electrodes (Unique Medical, Tokyo, Japan) were implanted via craniotomy in all patients. We selected ictal recordings using the following inclusion criteria: (1) preoperative magnetic resonance imaging (MRI) with neocortical lesions; (2) habitual seizures captured during IEEG recording; (3) no history of previous epilepsy surgery; (4) cases in which lesionectomy was performed; (5) seizure outcomes of Class 1a according to the International League Against Epilepsy (ILAE) classification; and (6) provision of informed consent for participation. Six patients (22 seizures total) met these criteria and were included in the study.

Before electrode implantation, all patients underwent three days of prolonged video scalp EEG monitoring with Neurofax (Nihon Kohden, Tokyo, Japan) using a 10–20 system and 500 Hz sampling rate. Each patient underwent a comprehensive evaluation, including a high-resolution 3.0 Tesla brain MRI, fluorodeoxyglucose positron emission tomography, and neuropsychological assessments. Experienced neuroradiologists and neurosurgeons evaluated the results of imaging studies. Intracranial electrode implantation was indicated when a more detailed evaluation was required or when discrepancies arose among ictal semiology, imaging, and EEG findings. This decision was made in a multidisciplinary meeting. Experienced neurosurgeons performed all epilepsy surgeries. Seizure outcomes were evaluated using the ILAE classification at the final confirmation after surgery (Table 1).

**Table 1:** Patient profiles.

Patient	Sex	Age at onset (years)	Age at surgery (years)	ASM	Ictal semiology	MRI lesion	Number of seizures	Pathology	Follow-up (months)	ILAE outcome
1	M	4	5	LEV	FAS, FBTCS	Rt. superior frontal gyrus, cingulate gyrus	2	FCD type IIb	73	1a
2	M	5	8	PB, PER, LCM	FAS, FBTCS	Lt. middle frontal gyrus	5	FCD type IIb	20	1a
3	F	12	15	CBZ, LEV, TPM	FIAS, FBTCS	Lt. inferior parietal lobule	4	FCD type IIb	24	1a
4	M	18	21	VPA, LEV, LCM	FAS, FIAS	Lt. fusiform gyrus	2	Ganglioglioma	35	1a
5	F	10	32	CBZ, LTG, LEV	FAS, FBTCS	Lt. superior parietal lobule	1	FCD type IIb	76	1a
6	M	30	35	CBZ, LEV	FAS, FBTCS	Lt. middle frontal gyrus	8	FCD type IIb	102	1a

ASM, anti-seizure medication; CBZ, carbamazepine; F, female; FAS, focal-aware seizure; FBTCS, focal-to-bilateral tonic–clonic seizure; FCD, focal cortical dysplasia; FIAS, focal impaired awareness seizure; ILAE, International League Against Epilepsy; LCM, lacosamide; LEV, levetiracetam; Lt, left; LTG, lamotrigine; M, male; MRI, magnetic resonance imaging; PB, phenobarbital; PER, perampanel; Rt. right; TPM, topiramate; VPA, valproic acid.

### 2.2 Intracranial electroencephalographic recording

**Figure 1:**
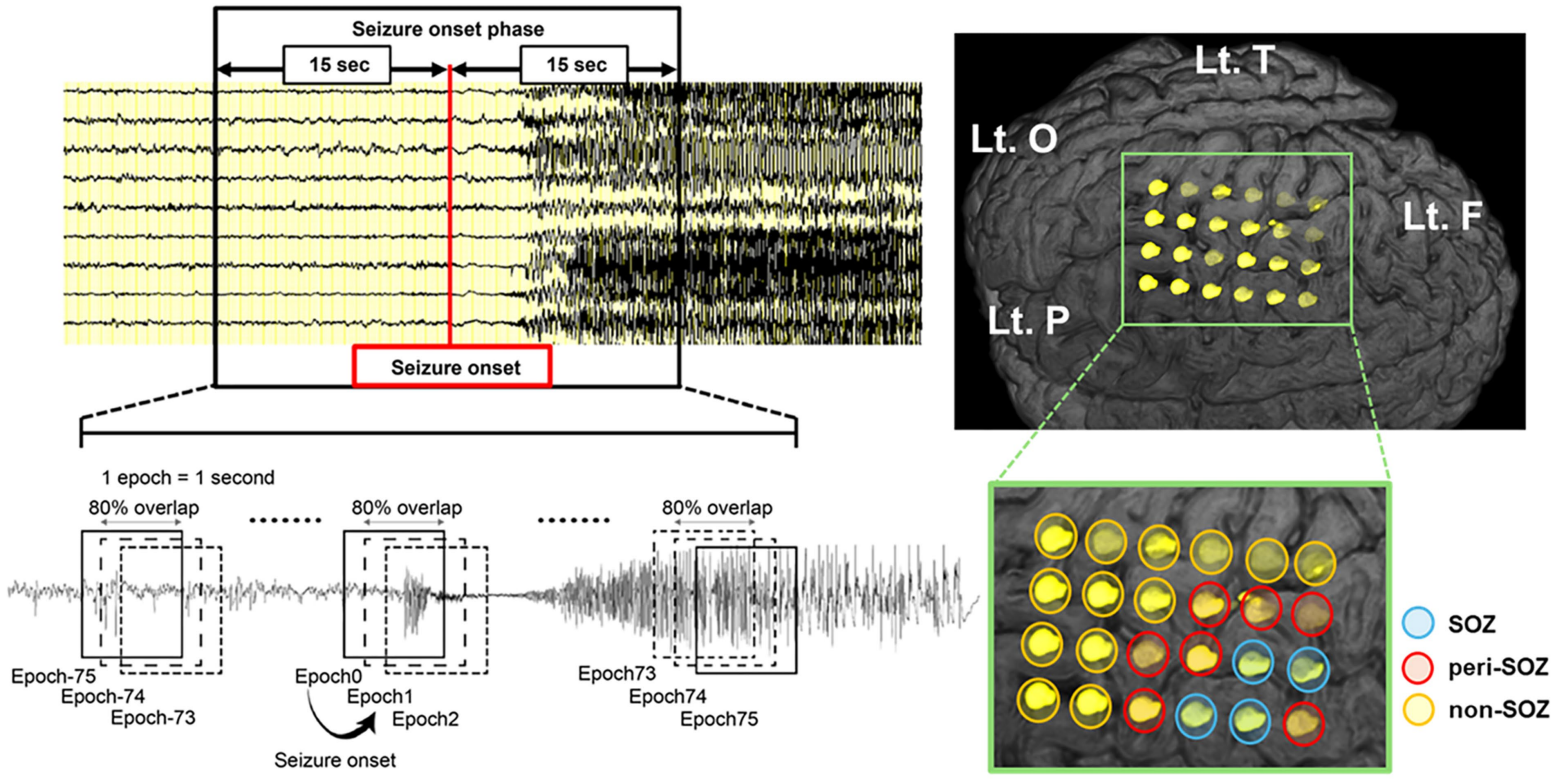
Schematic illustration of the sliding window technique for seizure onset phase and intracranial electrode placement in a representative case (Pt.4). Seizure onset was visually identified, and a 30 s window centered on the seizure onset was extracted. EEG data were segmented into 1 s epochs with 80% overlap using a sliding window technique. The modulation index was computed continuously for each electrode during the seizure onset phase. The SOZ (blue circle) was defined by visual inspection as the electrode contact that showed the first unequivocal ictal IEEG change. We defined peri-SOZ electrodes (red circle) as those adjacent to the SOZ electrodes and non-SOZ electrodes (orange circle) as those excluding both SOZ and peri-SOZ electrodes. EEG, electroencephalography; F, frontal lobe; Lt., left; O, occipital lobe; P, parietal lobe; SOZ, seizure onset zone; T, temporal lobe.

The IEEG was recorded at a sampling rate of 1,000 Hz. Anti-seizure medications were temporarily withdrawn during IEEG monitoring. Our IEEG data were acquired for 32.5–138 h (72 h on average) until a sufficient number of seizures and IEDs were obtained. Two senior epileptologists visually diagnosed all seizures and SOZ. Visual inspection defined seizure onset as the first unequivocal IEEG change from the background that led to a clear seizure discharge without returning to background activity (25). The SOZ was the electrode contact that showed the first unequivocal ictal IEEG change. The seizure onset patterns were defined according to the criteria described by Lagarde et al. (26). We defined peri-seizure onset zone (peri-SOZ) electrodes as those adjacent to the SOZ electrodes and non-seizure onset zone (non-SOZ) electrodes as those excluding both SOZ and peri-SOZ electrodes (Figure 1).

### 2.3 Analysis of phase-amplitude coupling

PAC was measured using the modulation index (MI) method, which was introduced for assessing coupling strength (11, 27). We calculated the MI of each electrode contact following the methodology specified by Ueda et al. (22). The complex analytic signal *z(n)* of a time series with *N* points is defined as:


$$z(n)=a_{h}(n)e^{\mathrm{i\phi l}(n)}(n=1,2,\ldots ,N)$$

where *a_h_(n)* represents the instantaneous amplitude of the high-frequency component, and *ϕ_l_(n)* denotes the instantaneous phase of the low-frequency component. Both components are extracted using a bandpass Butterworth filter followed by the Hilbert transform. The MI, quantifying the strength of PAC, is calculated as:


$$\mathrm{MI}=|\frac{1}{N}\sum_{n=1}^{N}z(n)|\;(n=1,2,\ldots ,N)$$

The IEEG data were sampled at 1,000 Hz, and the amplifier bandpass was set to 0.08–300 Hz. Prior to analysis, artifactual channels were excluded. All quantitative IEEG analyses were conducted using a common average reference.

PAC analysis was performed using a sliding window approach, in which the analysis window was set to 1 s and shifted with 80% overlap to capture temporal dynamics in coupling strength.

We followed previous studies and employed the amplitude of two high-frequency bands (ripples: 80–200 Hz, fast ripples [FRs]: 200–300 Hz) and the phase of four slow-wave bands (1–2 Hz, 2–3 Hz, 3–4 Hz and 4–8 Hz) for MI calculation (13, 22). Slow-wave components below 1 Hz were excluded from the PAC analysis due to the limited temporal resolution of the 1 s analysis window.

For the MI analysis, a 30 s segment of IEEG data centered on seizure onset was extracted, resulting in 151 overlapping epochs (Figure 1).

The seizure onset phase was the analysis period from −75 to +75 epochs. The period from epochs −75 to 0 was classified as the pre-seizure onset phase, whereas the period from epochs +1 to +75 was classified as the post-seizure onset phase.

### 2.4 Comparative analysis of modulation index dynamics across the seizure onset zone, peri-seizure onset zone, and non-seizure onset zone

We calculated MI by coupling the amplitude of two HFO bands with the phase of four slow-wave bands, totaling eight MI values (MI_Ripples/1–2 Hz_, MI_Ripples/2–3 Hz_, MI_Ripples/3–4 Hz_, MI_Ripples/4–8 Hz_, MI_FRs/1–2 Hz_, MI_FRs/2–3 Hz_, MI_FRs/3–4 Hz_, and MI_FRs/4–8 Hz_). For each, we compared MI dynamics across three regions (SOZ, peri-SOZ, and non-SOZ) and two temporal phases (pre- and post-seizure onset).

We further compared mean MI values during the post-seizure onset phase among the SOZ, peri-SOZ, and non-SOZ regions.

### 2.5 Seizure onset zone localization using modulation index values

To evaluate whether MI values could predict SOZ, we performed binomial logistic regression and generated receiver operating characteristic (ROC) curves using the mean MI during the post-seizure onset phase (epochs +1 to +75). The area under the curve (AUC) quantified localization performance, and the AUCs of the ROC curves were compared.

To evaluate the ability of MI values to distinguish the SOZ from the peri-SOZ specifically, we performed ROC analysis and excluded non-SOZ data. The AUC for each ROC curve was calculated and compared.

### 2.6 Statistical analysis

All analyses were conducted using SPSS Statistics (version 29.0, IBM Corporation, Armonk, NY, United States) and MATLAB R2022b (MathWorks, Natick, MA, United States). The MI values during the seizure onset phase were tested for normality using the Shapiro–Wilk test, confirming non-parametric distribution. For MI dynamics comparison, group differences among SOZ, peri-SOZ, and non-SOZ were analyzed at each epoch from −75 to +75 using the Kruskal–Wallis test (*p* < 0.05), followed by Mann–Whitney U tests (*p* < 0.001) with Bonferroni correction for multiple comparisons. Mean MI values for each region during the post-seizure onset phase were compared using the interquartile range method after removing outliers. Statistical comparisons were performed using the Kruskal–Wallis test (*p* < 0.05), followed by Mann–Whitney U tests (*p* < 0.001) with Bonferroni correction for multiple comparisons. We numerically compared the AUC values from MI-based ROC curve analyses to assess their effectiveness for SOZ estimation without performing statistical significance testing. A post hoc power analysis was conducted to assess the statistical power associated with the comparison of mean MI values and the ROC-based classification performance.

### 2.7 Ethical approval

This study was conducted as a retrospective analysis, with data collected using the opt-in and opt-out methods. Ethical approval for the study, including registration and analysis, was obtained from the Ethics Committee of Juntendo University (approval number: 16–163). Written informed consent for the collection and storage of clinical information was obtained from patients and their parents.

## 3 Results

### 3.1 Clinical profiles

Table 1 summarizes the clinical profiles of the six patients. The age at seizure onset ranged from 4 to 30 years (median, 11 years), and the age at surgery ranged from 5 to 35 years (median, 18 years). The total number of intracranial electrode contacts ranged from 40 to 104. Seizure duration varied from 16 to 428 s (median: 87 s). Seizure onset patterns were categorized as follows: low-voltage fast activity (LVFA) in 55% of seizures (12 seizures), slow waves or baseline shifts followed by LVFA in 18% (4 seizures), rhythmic slow spikes in 14% (3 seizures), sharp theta/alpha activity in 9% (2 seizures), and bursts of polyspikes followed by LVFA in 4% (1 seizure). LVFA was observed in 77% (17 seizures) of all seizures. Pathological examination revealed five cases of focal cortical dysplasia type IIb and one case of ganglioglioma. The postoperative follow-up period ranged from 20 to 102 months (median: 54 months). Postoperative seizure outcomes were classified as ILAE class 1a in all patients. Electrode locations, the number of electrodes, and resection rates for each case are provided in Supplementary Table 1.

### 3.2 Comparative analysis of modulation index dynamics across the seizure onset zone, peri-seizure onset zone, and non-seizure onset zone regions during the seizure onset phase

**Figure 2:**
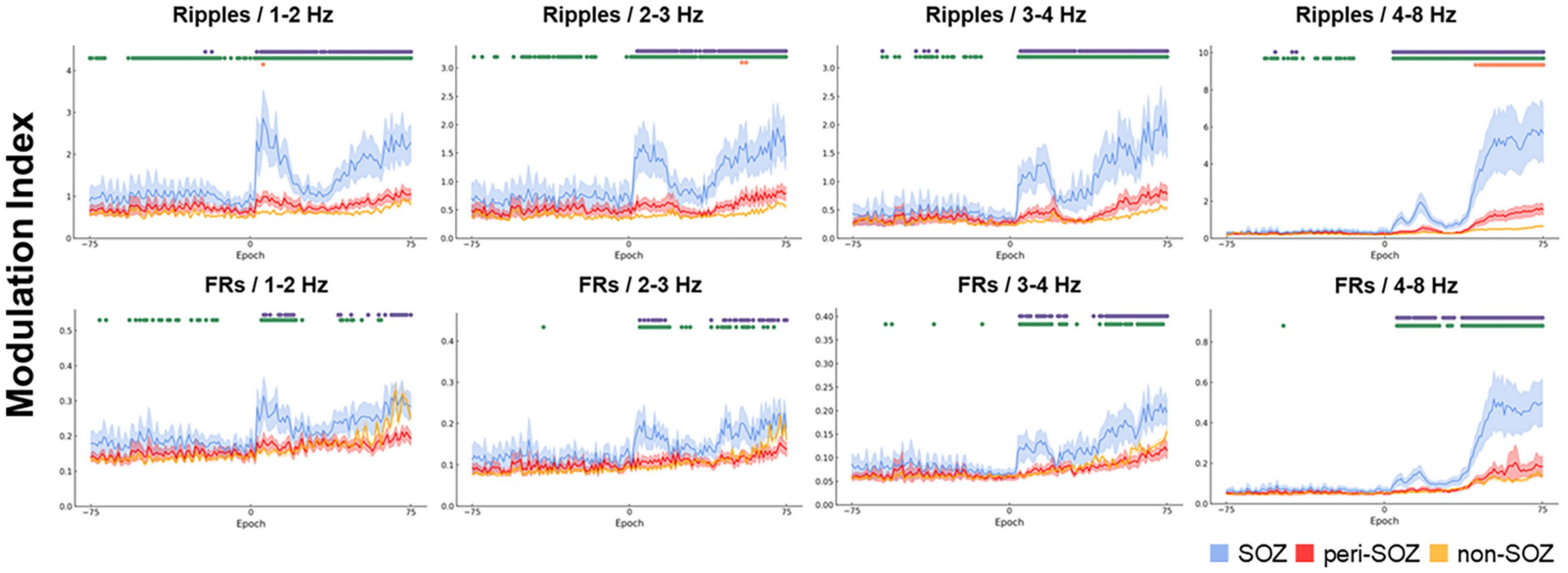
Comparative analysis of modulation index values across the seizure onset zone, peri-seizure onset zone, and non-seizure onset zone during the seizure onset phase. The temporal dynamics of the MIs for combinations of high-frequency oscillations and each slow-wave band are shown for a 30 s window centered on seizure onset (SOZ: light blue, peri-SOZ: red, non-SOZ: amber). The shaded areas for each waveform represent 95% confidence intervals. Markers displayed at the top of each graph represent epochs with significant differences in MI between regions. Purple indicates epochs in which the SOZ showed significantly higher MI than the peri-SOZ, green indicates epochs in which the SOZ showed higher MI than the non-SOZ, and orange indicates epochs in which the peri-SOZ showed higher MI than the non-SOZ. MI, modulation index; SOZ, seizure onset zone.

Figure 2 shows the MI dynamics during the seizure onset phase. For each epoch, we analyzed the MI values across SOZ, peri-SOZ, and non-SOZ regions. The results are categorized by region and phase.

#### 3.2.1 Seizure onset zone versus peri-seizure onset zone

During the pre-seizure onset phase, MI_Ripples/4–8 Hz_ in the SOZ was significantly higher than that in the peri-SOZ (*p* < 0.001) in 1.3% of epochs. MI_Ripples/3–4 Hz_ in the SOZ was significantly higher than that in the peri-SOZ (*p* < 0.001) in 3.9% of epochs. MI_Ripples/1–2 Hz_ in the SOZ was significantly higher than that in the peri-SOZ (*p* < 0.001) in 2.7% of epochs. MI_Ripples/2–3 Hz_ and MI_FRs/all slow waves_ showed no significant difference between the SOZ and peri-SOZ.

During the post-seizure onset phase, MI_Ripples/4–8 Hz_ in the SOZ was significantly higher than that in the peri-SOZ (*p* < 0.001) in 94.7% of epochs. In 92.0% of epochs during the post-seizure onset phase, MI_Ripples/3–4 Hz_ in the SOZ was significantly higher than that in the peri-SOZ (*p* < 0.001). In 89.3% of epochs during the post-seizure onset phase, MI_Ripples/2–3 Hz_ in the SOZ was significantly higher than that in the peri-SOZ (*p* < 0.001). In 93.3% of epochs during the post-seizure onset phase, MI_Ripples/1–2 Hz_ in the SOZ was significantly higher than that in the peri-SOZ (*p* < 0.001). In 81.3% of epochs during the post-seizure onset phase, MI_FRs/4–8 Hz_ in the SOZ was significantly higher than that in the peri-SOZ (*p* < 0.001). In 61.3% of epochs during the post-seizure onset phase, MI_FRs/3–4 Hz_ in the SOZ was significantly higher than that in the peri-SOZ (*p* < 0.001). In 41.3% of epochs during the post-seizure onset phase, MI_FRs/2–3 Hz_ in the SOZ was significantly higher than that in the peri-SOZ (*p* < 0.001). In 33.3% of epochs during the post-seizure onset phase, MI_FRs/1–2 Hz_ in the SOZ was significantly higher than that in the peri-SOZ (*p* < 0.001).

#### 3.2.2 Seizure onset zone versus non-seizure onset zone

During the pre-seizure onset phase, MI_Ripples/4–8 Hz_ in the SOZ was significantly higher than that in the non-SOZ (*p* < 0.001) in 28.9% of epochs. In 18.4% of epochs during the pre-seizure onset phase, MI_Ripples/3–4 Hz_ in the SOZ was significantly higher than that in the non-SOZ (*p* < 0.001). In 46.7% of epochs during the pre-seizure onset phase, MI_Ripples/2–3 Hz_ in the SOZ was significantly higher than that in the non-SOZ (*p* < 0.001). In 74.4% of epochs during the pre-seizure onset phase, MI_Ripples/3–4 Hz_ in the SOZ was significantly higher than that in the non-SOZ (*p* < 0.001). In 3.9% of epochs during the pre-seizure onset phase, MI_FRs/3–4 Hz_ in the SOZ was significantly higher than that in the non-SOZ (*p* < 0.001). In 1.3% of epochs during the pre-seizure onset phase, MI_FRs/2–3 Hz_ in the SOZ was significantly higher than that in the non-SOZ (*p* < 0.001). In 28.0% of epochs during the pre-seizure onset phase, MI_FRs/1–2 Hz_ in the SOZ was significantly higher than that in the non-SOZ (*p* < 0.001). MI_FRs/4–8 Hz_ showed no significant difference between the SOZ and non-SOZ regions.

During the post-seizure onset phase, MI_Ripples/4–8 Hz_ in the SOZ was significantly higher than that in the non-SOZ (*p* < 0.001) in 96.0% of epochs. In 94.7% of epochs during the post-seizure onset phase, MI_Ripples/3–4 Hz_ in the SOZ was significantly higher than that in the non-SOZ (*p* < 0.001). In 98.7% of epochs during the post-seizure onset phase, MI_Ripples/2–3 Hz_ in the SOZ was significantly higher than that in the non-SOZ (*p* < 0.001). In 98.7% of epochs during the post-seizure onset phase, MI_Ripples/1–2 Hz_ in the SOZ was significantly higher than that in the non-SOZ (*p* < 0.001). In 81.3% of epochs during the post-seizure onset phase, MI_FRs/4–8 Hz_ in the SOZ was significantly higher than that in the non-SOZ (*p* < 0.001). In 60.0% of epochs during the post-seizure onset phase, MI_FRs/3–4 Hz_ in the SOZ was significantly higher than that in the non-SOZ (*p* < 0.001). In 45.3% of epochs during the post-seizure onset phase, MI_FRs/2–3 Hz_ in the SOZ was significantly higher than that in the non-SOZ (*p* < 0.001). In 34.7% of epochs during the post-seizure onset phase, MI_FRs/1–2 Hz_ in the SOZ was significantly higher than that in the non-SOZ (*p* < 0.001).

#### 3.2.3 Peri-seizure onset zone versus non-seizure onset zone

During the pre-seizure onset phase, no MI values in the peri-SOZ were significantly higher than those in the non-SOZ.

In 42.7% of epochs during the post-seizure onset phase, MI_Ripples/4–8 Hz_ in the peri-SOZ was significantly higher than that in the non-SOZ (*p* < 0.001). In 2.7% of epochs during the post-seizure onset phase, MI_Ripples/2–3 Hz_ in the peri-SOZ was significantly higher than that in the non-SOZ (*p* < 0.001). In 1.3% of epochs during the post-seizure onset phase, MI_Ripples/1–2 Hz_ in the peri-SOZ was significantly higher than that in the non-SOZ (*p* < 0.001). The other MI values in the peri-SOZ did not show significantly higher values compared to the non-SOZ.

### 3.3 Comparison of mean modulation index values during the post-seizure onset phase

**Figure 3:**
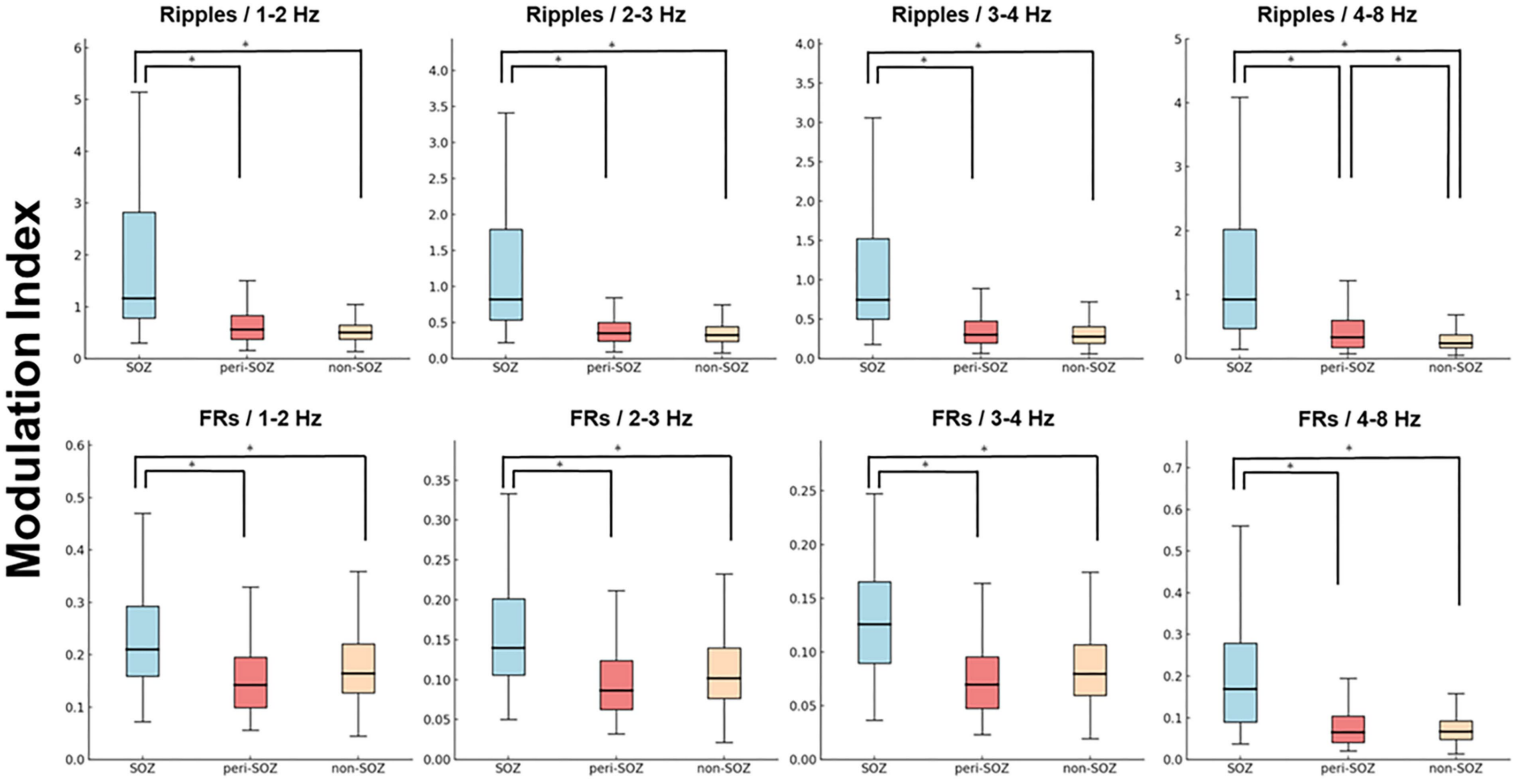
Comparison of mean modulation index values during the post-seizure onset phase (epochs 1–75). Box plot showing MI values from epochs 1 to 75 in the SOZ, peri-SOZ, and non-SOZ. Statistical significance was determined using the Kruskal–Wallis test, followed by Mann–Whitney U tests for pairwise comparisons. Statistical significance was set at *p* < 0.001, with Bonferroni correction for multiple comparisons. The SOZ exhibited significantly higher MI values than peri-SOZ and non-SOZ regions across all MI combinations. In the MI_Ripples/4–8 Hz_ analysis, the peri-SOZ showed significantly higher values than the non-SOZ [asterisk (*) represents *p* < 0.001]. FRs, fast ripples; MI, modulation index; SOZ, seizure onset zone.

Figure 3 shows a box plot of mean MI values across SOZ, peri-SOZ, and non-SOZ regions during the post-seizure onset phase.

MI_Ripples/4–8 Hz_ in the SOZ was significantly higher than that in the peri-SOZ and non-SOZ regions (*p* < 0.0001 for both comparisons). MI_Ripples/4–8 Hz_ in the peri-SOZ was significantly higher than that in the non-SOZ (*p* < 0.0001). MI_Ripples/3–4 Hz_ in the SOZ was significantly higher than that in the peri-SOZ and non-SOZ (*p* < 0.0001 for both comparisons). No significant differences were observed between the peri-SOZ and non-SOZ (*p* = 0.48). MI_Ripples/2–3 Hz_ in the SOZ was significantly higher than that in the peri-SOZ and non-SOZ (*p* < 0.0001 for both comparisons). No significant differences were observed between the peri-SOZ and non-SOZ (*p* = 0.086). MI_Ripples/1–2 Hz_ in the SOZ was significantly higher than that in the peri-SOZ and non-SOZ (*p* < 0.0001 for both comparisons). No significant differences were observed between the peri-SOZ and non-SOZ (*p* = 0.19).

MI_FRs/4–8 Hz_ in the SOZ was significantly higher than that in the peri-SOZ and non-SOZ (*p* < 0.0001 for both comparisons). However, no significant differences were observed between the peri-SOZ and non-SOZ (*p* = 1.00). MI_FRs/3–4 Hz_ in the SOZ was significantly higher than that in the peri-SOZ and non-SOZ (*p* < 0.0001 for both comparisons). No significant differences were observed between the peri-SOZ and non-SOZ (*p* = 0.034). MI_FRs/2–3 Hz_ in the SOZ was significantly higher than that in the peri-SOZ and non-SOZ (*p* < 0.0001 for both comparisons). No significant differences were observed between the peri-SOZ and non-SOZ (*p* = 0.29). MI_FRs/1–2 Hz_ in the SOZ was significantly higher than that in the peri-SOZ and non-SOZ (*p* < 0.0001 for both comparisons). No significant differences were observed between the peri-SOZ and non-SOZ (*p* = 0.027).

### 3.4 Seizure onset zone localization using the modulation index

**Figure 4:**
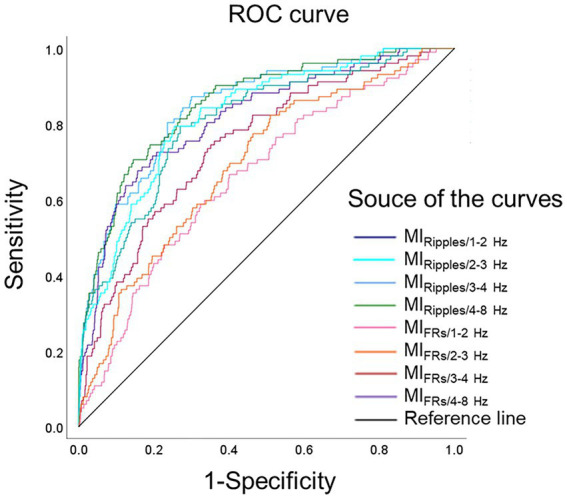
Receiver operating characteristics curves for localization of the seizure onset zone. ROC curves for localization of seizure onset zone, using MI_Ripples/1–2 Hz_ (deep blue line), MI_Ripples/2–3 Hz_ (light blue line), MI_Ripples/3–4 Hz_ (blue line), MI_Ripples/4–8 Hz_ (green line), MI_FRs/1–2 Hz_ (pink line), MI_FRs/2–3 Hz_ (orange line), MI_FRs/3–4 Hz_ (red line), MI_FRs/4–8 Hz_ (purple line), and reference line is black. MI_Ripples/4–8 Hz_ show an AUC of 0.853 (*p* < 0.001), MI_Ripples/3–4 Hz_ show an AUC of 0.846 (*p* < 0.001), MI_Ripples/2–3 Hz_ show an AUC of 0.822 (*p* < 0.001), and MI_FRs/4–8 Hz_ show an AUC of 0.815 (*p* < 0.001). AUC, area under the curve; FRs, fast ripples; MI, modulation index; ROC, receiver operating characteristics.

Figure 4 shows the ROC curves for localization of SOZ using the mean MI values during the post-seizure onset phase. Table 2 summarizes the results of the ROC curve analysis for SOZ localization. In the ROC curve analysis for SOZ localization, MI_Ripples/4–8 Hz_, MI_Ripples/3–4 Hz_, MI_Ripples/2–3 Hz,_ MI_FRs/4–8 Hz_ and MI_Ripples/1–2 Hz_ showed AUC values of 0.853, 0.846, 0.822, 0.815, and 0.806, respectively (all *p* < 0.001).

**Table 2:** Receiver operating characteristic analysis for the performance of modulation index in localizing seizure onset zone.

Predictor	AUC	95% CI lower	95% CI upper	*p*-value	Sensitivity	Specificity
MI_Ripples/1–2 Hz_	0.806	0.761	0.851	<0.001	0.794	0.730
MI_Ripples/2–3 Hz_	0.822	0.781	0.863	<0.001	0.794	0.745
MI_Ripples/3–4 Hz_	0.846	0.807	0.885	<0.001	0.804	0.764
MI_Ripples/4–8 Hz_	0.853	0.814	0.892	<0.001	0.745	0.805
MI_FRs/1–2 Hz_	0.662	0.607	0.716	<0.001	0.667	0.599
MI_FRs/2–3 Hz_	0.694	0.642	0.747	<0.001	0.824	0.489
MI_FRs/3–4 Hz_	0.747	0.698	0.797	<0.001	0.696	0.669
MI_FRs/4–8 Hz_	0.815	0.769	0.861	<0.001	0.725	0.774

This table summarizes the ROC curves and AUC values for each predictor, highlighting their performance in localizing the seizure onset zone. Larger AUC values indicate better localization ability. A 95% CI of the AUC is provided for each predictor variable. The sensitivity and specificity values are shown at the point where the sum is maximized. AUC, area under the curve; CI, confidence interval; FRs, fast ripples; MI, modulation index; ROC, receiver operating characteristic; SOZ, seizure onset zone.

### 3.5 Differentiating the seizure onset zone from the peri-seizure onset zone using modulation index values

**Figure 5:**
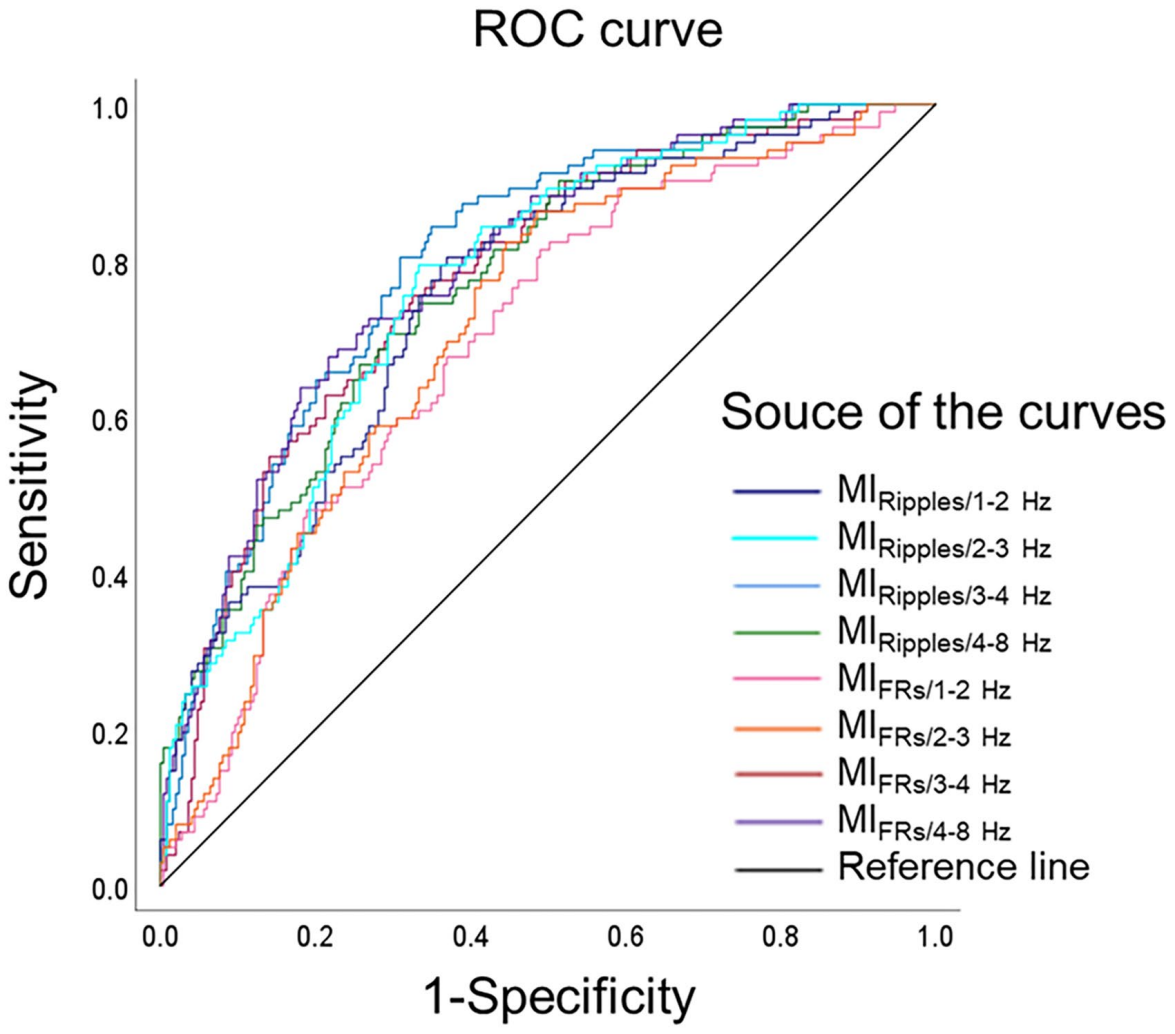
Receiver operating characteristics curves for differentiating the seizure onset zone. ROC curves for differentiating the SOZ from the peri-SOZ, using MI_Ripples/1–2 Hz_ (deep blue line), MI_Ripples/2–3 Hz_ (light blue line), MI_Ripples/3–4 Hz_ (blue line), MI_Ripples/4–8 Hz_ (green line), MI_FRs/1–2 Hz_ (pink line), MI_FRs/2–3 Hz_ (orange line), MI_FRs/3–4 Hz_ (red line), MI_FRs/4–8 Hz_ (purple line), and reference line is black. MI_Ripples/3–4 Hz_ show an AUC of 0.800 (*p* < 0.001), MI_FRs/4–8 Hz_ show an AUC of 0.791 (*p* < 0.001), MI_FRs/3–4 Hz_ show an AUC of 0.775 (*p* < 0.001), and MI_Ripples/4–8 Hz_ shows an AUC of 0.771 (*p* < 0.001). AUC, area under the curve; FRs, fast ripples; MI, modulation index; ROC, receiver operating characteristics; SOZ, seizure onset zone.

Figure 5 shows ROC curves for differentiating SOZ from peri-SOZ using the mean MI values. Table 3 summarizes the ROC curve analysis results for differentiating between SOZ and peri-SOZ.

**Table 3:** Receiver operating characteristic analysis for assessing the performance of modulation index in differentiating the seizure onset zone from the peri-seizure onset zone.

Predictor	AUC	95% CI lower	95% CI upper	*p*-value	Sensitivity	Specificity
MI_Ripples/1–2 Hz_	0.756	0.702	0.810	<0.001	0.804	0.631
MI_Ripples/2–3 Hz_	0.767	0.716	0.819	<0.001	0.794	0.667
MI_Ripples/3–4 Hz_	0.800	0.751	0.848	<0.001	0.804	0.691
MI_Ripples/4–8 Hz_	0.771	0.718	0.823	<0.001	0.706	0.707
MI_FRs/1–2 Hz_	0.696	0.637	0.755	<0.001	0.814	0.510
MI_FRs/2–3 Hz_	0.712	0.655	0.769	<0.001	0.824	0.554
MI_FRs/3–4 Hz_	0.775	0.722	0.827	<0.001	0.696	0.707
MI_FRs/4–8 Hz_	0.791	0.741	0.841	<0.001	0.725	0.719

This table summarizes the ROC curves and corresponding AUC values for each predictor, highlighting their effectiveness in distinguishing the SOZ from the peri-SOZ region. Larger AUC values indicated superior differentiation capability. A 95% CI of the AUC is provided for each predictor variable. The sensitivity and specificity values are presented at the point where the combined sum is maximized. AUC, area under the curve; CI, confidence interval; FRs, fast ripples; MI, modulation index; ROC, receiver operating characteristic; SOZ, seizure onset zone.

In the ROC curve analysis for differentiating the SOZ from peri-SOZ, MI_Ripples/3–4 Hz_, MI_FRs/4–8 Hz_, MI_FRs/3–4 Hz_, and MI_Ripples/4–8 Hz_ showed AUC values of 0.800, 0.791, 0.775, and 0.771, respectively (all *p* < 0.001).

### 3.6 Post hoc evaluation of statistical power

We performed post hoc power analyses, as summarized in Supplementary Tables 2, 3, to evaluate the reliability of statistical comparisons (Result section 3.3) and ROC analyses (Result section 3.4 and 3.5). A total of 48 MI comparisons were assessed, including 24 for MI_Ripples/slow waves_ and 24 for MI_FRs/slow waves_. Among these, 23 of 24 comparisons for MI_Ripples/slow waves_ and 20 of 24 for MI_FRs/slow waves_ demonstrated sufficient statistical power (power > 0.8). The five comparisons with lower power all corresponded to non-significant results reported in Result section 3.3.

## 4 Discussion

### 4.1 Summary of findings

This study investigated PAC in intracranial EEG to localize SOZ in patients with drug-resistant neocortical epilepsy. We analyzed 22 seizures from six patients and computed the MI using a sliding window technique over a 30 s window centered on seizure onset. MI values were significantly higher in the SOZ than in the peri-SOZ and non-SOZ regions, particularly during the post-seizure onset phase. MI_Ripples/4–8 Hz_ in the peri-SOZ was significantly higher than that in the non-SOZ during the post-seizure onset phase. MI_Ripples/4–8 Hz_ exhibited the ability to localize the SOZ (AUC 0.853). MI_Ripples/3–4 Hz_ exhibited the ability to discriminate the SOZ from the peri-SOZ (AUC 0.800). These findings suggest that MI analysis during the seizure onset phase may be a biomarker for identifying SOZ in the surrounding brain regions.

### 4.2 Notability of this study

A key novelty of the present study lies in its detailed classification of electrode contacts into SOZ, peri-SOZ, and non-SOZ regions at the intracranial electrode level. Whereas prior studies commonly classified peri-SOZ and non-SOZ electrodes into a single group, our method enables finer differentiation of ictal activity patterns in each respective region.

We demonstrated that ictal MI dynamics during the seizure onset phase differ across the SOZ, peri-SOZ, and non-SOZ regions, with statistically significant differences in MI values across three regions. Refined slow-wave band segmentation facilitated detailed analysis of ictal MI dynamics, which exhibited coupling-dependent temporal profiles across regions.

In addition, we further assessed whether ictal PAC could quantitatively distinguish the SOZ from peri-SOZ using ROC curve analysis. Building upon prior studies that combined peri-SOZ and non-SOZ electrodes into a single group, our approach offered a more structured framework for differentiating the SOZ from adjacent contacts with higher spatial resolution. This electrode contact-level evaluation, supported by ROC-based metrics, may provide additional insights into SOZ localization and support the use of ictal PAC as a complementary biomarker for refining surgical strategy in neocortical epilepsy.

### 4.3 Dynamics of modulation index in the seizure onset zone and peri-seizure onset zone during the seizure onset phase

In this study, we demonstrated the temporal dynamics of MI during the seizure onset phase of neocortical epilepsy. The MI values in the SOZ significantly increased after seizure onset compared with those in the peri-SOZ and non-SOZ regions, particularly in MI_Ripples/4–8 Hz_ and MI_Ripples/3–4 Hz_.

Only few studies have been conducted on ictal PAC. In temporal lobe epilepsy, ictal PAC in EZ shows different dynamics depending on the combination of HFOs and slow waves (22). In this study, MI values rapidly increased during the preictal spiking phase regardless of the HFO–slow-wave combination. MI _Ripples/3–4 Hz_ displayed a dip-shaped pattern, showing a temporary decrease during the LVFA phase before rising again. In contrast, MI_Ripples/4–8 Hz_ showed a sustained increase from seizure onset, peaked, and subsequently declined toward seizure termination. Regardless of seizure type or EZ anatomical location, the PAC between HFOs (ripples/FRs) and delta/theta waves increases at seizure onset contacts during seizure generation (18). Additionally, the PAC between ripples and theta waves increases during a seizure and reaches its peak earlier than the PAC between the ripples and delta waves, which gradually increases throughout the seizure and peaks later (19).

Our findings in neocortical epilepsy align with previous reports (18, 19, 22), showing elevated MI values within the SOZ during the post-seizure onset phase. Additionally, our findings confirmed that ictal MI dynamics varies across the SOZ, peri-SOZ, and non-SOZ regions. The MI_Ripples/4–8 Hz_ in the SOZ was particularly prominent, began to increase from the onset, exhibited a transient decrease, and rose sharply. MI_Ripples/4–8 Hz_ in the peri-SOZ also increased during the late post-seizure onset phase, becoming significantly higher than that in the non-SOZ. These findings suggest that PAC between ripples and 4–8 Hz waves may serve as a driving force for seizure activity, contributing to seizure generation and early propagation.

Furthermore, MI_Ripples/1–2 Hz_, MI_Ripples/2–3 Hz_, and MI_Ripples/3–4 Hz_ were significantly higher in the SOZ than in the peri-SOZ and non-SOZ during the post-seizure onset phase. MI_Ripples/1–2 Hz_, MI_Ripples/2–3 Hz_, and MI_Ripples/3–4 Hz_ in the SOZ showed a steep increase immediately after seizure onset, followed by a transient decrease and a gradual increase. In contrast, MI_Ripples/1–2 Hz_, MI_Ripples/2–3 Hz_, and MI_Ripples/3–4 Hz_ in the peri-SOZ and non-SOZ exhibited a slower and more gradual increase, with minimal to no significant differences observed between these regions. The role of PAC between ripples and delta (1–2 Hz, 2–3 Hz and 3–4 Hz) waves in seizure generation remains unclear, but their involvement in early seizure propagation may be limited compared with that of PAC between ripples and 4–8 Hz waves.

At seizure onset, PAC between ripples and 4–8 Hz waves is speculated to increase and may act as a driving force for seizure initiation. In contrast, PAC between ripples and delta (1–2 Hz, 2–3 Hz and 3–4 Hz) waves within the SOZ may serve as a negative feedback mechanism to suppress seizure activity. When this inhibitory control fails to counterbalance excitation, the PAC between ripples and 4–8 Hz waves may increase further, contributing to seizure progression. Subsequently, a secondary increase in PAC between ripples and delta (1–2 Hz, 2–3 Hz and 3–4 Hz) waves may emerge, potentially playing a role in seizure termination.

### 4.4 Identification and differentiation of the seizure onset zone from the peri-seizure onset zone using modulation index analysis during the seizure onset phase

To our knowledge, this is the first study to differentiate the SOZ from peri-SOZ regions at each electrode site using ictal MI dynamics in neocortical epilepsy. ROC analysis demonstrated that MI_Ripples/4–8 Hz_ was the most accurate metric for SOZ estimation, while MI_Ripples/3–4 Hz_ provided the highest accuracy in differentiating SOZ from peri-SOZ. We speculated that PAC between ripples and 4–8 Hz waves is involved in seizure onset and propagation and an increase in propagation regions, contributing to the estimation of higher epileptogenicity and possibly reducing the discrimination accuracy between SOZ and peri-SOZ. PAC between ripples and 3–4 Hz waves increased in the SOZ but did not exhibit early propagation to the peri-SOZ, which may have enhanced the accuracy of differentiating the SOZ from surrounding regions. Peri-SOZ regions exhibiting increased MI_Ripple/4–8 Hz_ may reflect areas involved in early seizure propagation. These regions could be considered potential targets for resection.

Various studies have highlighted the utility of computational analysis of IEEG for identifying the EZ. Several methods, including HFO occurrence rate and amplitude (9), interictal spikes (8, 28), relative entropy (10), and PAC (12, 29), have been proposed for interictal analysis. A previous study using network analysis of interictal spikes reported differences between the resected region, surrounding margin zone, and non-EZ regions (30) but did not analyze localization based on individual electrode data.

Fewer approaches have been reported for ictal analysis than for interictal analysis. These include ictal HFOs (31, 32), Epileptogenicity Index (33), slow polarizing shift index (34), and phase transfer entropy (35).

Ma et al. (24) localized the EZ using PAC analysis of interictal and ictal data by applying a slow-wave phase (0.5–10 Hz), fast oscillation amplitude (13–200 Hz), and a 10 s time window. They reported that interictal PAC yielded more accurate EZ localization than ictal PAC during the early seizure phase, suggesting its potential as a reliable biomarker. While interictal PAC demonstrated higher accuracy, ictal PAC analysis during the seizure onset phase may also contribute to accurate localization of the SOZ, particularly when analytical parameters such as frequency selection and time window design are carefully optimized. PAC between slow waves and HFOs may have different functional roles depending on the frequency band of the slow waves (17, 19). These findings indicate that analyzing PAC by separating slow-wave phase bands may help reveal their distinct contributions to seizure dynamics.

In this study, we applied a sliding window technique with a 1 s window and conducted PAC analysis across each phase of the slow-wave band. We enhanced temporal resolution and captured dynamic changes over time by shortening the window and introducing an overlap. This approach revealed distinct MI dynamics between HFOs and slow waves across the SOZ, peri-SOZ, and non-SOZ regions, enabling the identification of frequency combinations closely associated with seizure generation. These PAC patterns likely contribute to the high accuracy of SOZ localization by reflecting the underlying seizure onset mechanisms.

### 4.5 Limitations

This study has some limitations. First, owing to the strict inclusion criteria, the sample size was small, consisting of 22 seizures from six patients. To assess the statistical strength of our findings, we conducted post hoc power analyses (Supplementary Tables 2, 3). In analyses based on individual electrode contacts, most comparisons with statistically significant differences demonstrated sufficient power (power > 0.8), which is generally considered an acceptable threshold for detecting group differences. However, a few comparisons showed insufficient power, these findings should be interpreted with caution. In the future, accumulating more cases will likely be necessary to facilitate further analysis.

Second, because intracranial electrodes only cover specific brain regions, the true SOZ is not guaranteed to be fully covered. This indicates that the activity observed in the estimated SOZ may not fully correspond to the true SOZ activity. The patients included in this study had identifiable lesions on imaging that were sufficiently covered by electrodes (subdural grid and/or depth), and all achieved seizure freedom after lesionectomy. Except for cases where seizure onset originated in deep brain structures, most of the SOZ is likely to have been adequately covered.

Third, due to the 1 kHz sampling rate used in this study, FRs analysis was restricted to the 200–300 Hz frequency range. Previous studies have shown that ictal FRs in the 300–500 Hz or >1,000 Hz range may localize to the SOZ and correlate with favorable surgical outcomes (32, 36). FRs power has been reported to increase during seizures (37). However, prior PAC studies with a 1 kHz sampling rate defined FRs as the 200/250–300 Hz and reported significant associations with epileptogenicity (13, 38). Moreover, the spectral frequency of neocortical HFOs has been shown to mostly fall below 300 Hz (39, 40). Although the analysis of FRs was limited to the 200–300 Hz range, our finding of elevated MI_FRs/slow waves_ in the post seizure onset phase is consistent with the previous report (37). We believe the present analysis remains valid and informative, though future studies with higher sampling rates are warranted.

Additionally, we examined ictal PAC using a sliding window technique with overlapping analyses. This approach involves methodological considerations regarding the selection of an appropriate window size. If the window is too short, sufficient data for reliable PAC estimation may not be captured, particularly when assessing the slow-wave activity phase. In contrast, transient yet meaningful changes may be obscured if the window is too long. To balance these considerations and ensure an adequate temporal resolution, we selected a window size of one second. While this setting enabled the capture of rapid PAC fluctuations, it limited the analysis of slower oscillatory components, especially those below 1 Hz. Slow oscillations, such as direct current shifts and infraslow activity, can emerge in the SOZ during seizures (41); these components were not examined in detail in the present study. Their potential role in coupling with HFOs warrants further investigation in ictal PAC.

Finally, the high prevalence of FCD type IIb may have influenced our findings. Using a different methodology, Ricci et al. (42) reported stronger ictal PAC in FCD type IIb than in FCD I with no significant differences between SOZ and the others in type I, suggesting pathology-specific effects on PAC that were not explored in this study. Further research is needed to clarify how pathology influences ictal PAC dynamics within our framework.

## 5 Conclusion

A significant increase in MI values, particularly between ripples and slow waves, was observed in the SOZ during the seizure onset phase. MI_Ripples/4–8 Hz_ demonstrated strong performance in localizing the SOZ, and MI_Ripples/3–4 Hz_ distinguished the SOZ from peri-SOZ. These findings suggest that ictal PAC analysis may enhance the accuracy of presurgical evaluations and delineate resection margins. Ictal PAC analysis may be a valuable biomarker for localization of the SOZ in drug-resistant neocortical epilepsy.

## Data Availability

The raw data supporting the conclusions of this article will be made available by the authors, without undue reservation.
