# Supplementary material for: Ictal phase-amplitude coupling as a biomarker for seizure onset zone in neocortical epilepsy
Source: Front Neurol. 2025 Sep 3;16:1632484. doi: 10.3389/fneur.2025.1632484 (PMC12440772; doi:10.3389/fneur.2025.1632484)
Supplement: Supplementary file 1 [file Table_1.DOCX]

Supplementary data (Table 1, Table 2, Table 3)

| **Table S1. Location and number of electrocorticography (ECoG) electrodes and resection rate of SOZ, peri-SOZ and non-SOZ.** | | | | | | | | | |  |
| --- | --- | --- | --- | --- | --- | --- | --- | --- | --- | --- |
| Patient | ECoG electrode location | Number of electorde contact | | | | |  | resection rate | | |
|  |  | Total number | SOZ | peri-SOZ | non-SOZ | bad electrode | EZ | SOZ | peri-SOZ | non-SOZ |
| 1 | Rt. F | 42 | 3 | 12 | 26 | 1 | 7 | 100.0 | 33.3 | 0.0 |
| 2 | Lt. F | 40 | 5 | 8 | 26 | 1 | 14 | 100.0 | 37.5 | 23.1 |
| 3 | Lt. F, T, P | 88 | 15 | 20 | 25 | 28 | 6 | 0.0 | 20.0 | 8.0 |
| 4 | Lt. F, T, P | 104 | 2 | 4 | 96 | 2 | 4 | 100.0 | 50.0 | 0.0 |
| 5 | Lt. F, T, P | 78 | 4 | 8 | 57 | 9 | 15 | 75.0 | 50.0 | 12.3 |
| 6 | Lt. F, T | 44 | 3 | 12 | 28 | 1 | 14 | 100.0 | 58.3 | 14.3 |

Abbreviations: EZ, epileptogenic zone; F, frontal lobe; Lt., left; P, parietal lobe; Rt., right; SOZ, seizure onset zone; T, temporal lobe

bad electrode: excluded due to artifactual signals; EZ: Electrodes that were actually resected.

As shown in the table, Patients No. 1, 2, 4, 5, and 6 demonstrated a high rate of SOZ resection. In Patient No. 3, the SOZ was located in the eloquent cortex surrounding the lesion. We resected the lesion while preserving the eloquent areas, resulting in seizure freedom for 24 months.

| **Table S2. Post hoc power analysis for comparion of MI_Ripples/slow waves_ across SOZ, peri-SOZ, non-SOZ.** | | | |  |  |  |
| --- | --- | --- | --- | --- | --- | --- |
| MI_HFOs/slow waves_ | Comparison | Statistical test | Effect size (Cohen’s d / η²) | Sample size(n/group) | α level | Power (1–β) |
|  |  |  |  |  |  |  |
| MI_Ripples/1-2 Hz_ | SOZ vs. peri-SOZ vs non-SOZ | Kruskal–Wallis test | 0.096(η^2^) | 102 vs. 249 vs. 784 | 0.05 | 1 |
|  | SOZ vs. peri-SOZ | Mann–Whitney U test | 0.841 (Cohen’s d) | 102 vs. 249 | 0.05 | 1 |
|  | SOZ vs. non-SOZ | Mann–Whitney U test | 1.116 (Cohen’s d) | 102 vs. 784 | 0.05 | 1 |
|  | peri-SOZ vs. non-SOZ | Mann–Whitney U test | 0.322 (Cohen’s d) | 249 vs. 784 | 0.05 | 0.948 |
|  | SOZ localization (SOZ vs. peri-&non-SOZ) | Binominal logistic regression analysis | 0.690(Cohen’s d) | 102 vs. 1033 | 0.05 | 1 |
|  | SOZ localization (SOZ vs. peri-SOZ) | Binominal logistic regression analysis | 0.473(Cohen’s d) | 102 vs. 249 | 0.05 | 1 |
| MI_Ripples/2-3 Hz_ | SOZ vs. peri-SOZ vs non-SOZ | Kruskal–Wallis test | 0.108(η^2^) | 102 vs. 249 vs. 784 | 0.05 | 1 |
|  | SOZ vs. peri-SOZ | Mann–Whitney U test | 0.806 (Cohen’s d) | 102 vs. 249 | 0.05 | 1 |
|  | SOZ vs. non-SOZ | Mann–Whitney U test | 1.102 (Cohen’s d) | 102 vs. 784 | 0.05 | 1 |
|  | peri-SOZ vs. non-SOZ | Mann–Whitney U test | 0.358 (Cohen’s d) | 249 vs. 784 | 0.05 | 0.979 |
|  | SOZ localization (SOZ vs. peri-&non-SOZ) | Binominal logistic regression analysis | 0.912(Cohen’s d) | 102 vs. 1033 | 0.05 | 1 |
|  | SOZ localization (SOZ vs. peri-SOZ) | Binominal logistic regression analysis | 0.595(Cohen’s d) | 102 vs. 249 | 0.05 | 1 |
| MI_Ripples/3-4 Hz_ | SOZ vs. peri-SOZ vs non-SOZ | Kruskal–Wallis test | 0.1207(η^2^) | 102 vs. 249 vs. 784 | 0.05 | 1 |
|  | SOZ vs. peri-SOZ | Mann–Whitney U test | 0.856 (Cohen’s d) | 102 vs. 249 | 0.05 | 0.87 |
|  | SOZ vs. non-SOZ | Mann–Whitney U test | 1.124 (Cohen’s d) | 102 vs. 784 | 0.05 | 0.81 |
|  | peri-SOZ vs. non-SOZ | Mann–Whitney U test | 0.322 (Cohen’s d) | 249 vs. 784 | 0.05 | 0.42 |
|  | SOZ localization (SOZ vs. peri-&non-SOZ) | Binominal logistic regression analysis | 1.055(Cohen’s d) | 102 vs. 1033 | 0.05 | 1 |
|  | SOZ localization (SOZ vs. peri-SOZ) | Binominal logistic regression analysis | 0.689(Cohen’s d) | 102 vs. 249 | 0.05 | 1 |
| MI_Ripples/4-8 Hz_ | SOZ vs. peri-SOZ vs non-SOZ | Kruskal–Wallis test | 0.1474(η^2^) | 102 vs. 249 vs. 784 | 0.05 | 1 |
|  | SOZ vs. peri-SOZ | Mann–Whitney U test | 0.782(Cohen’s d) | 102 vs. 249 | 0.05 | 1 |
|  | SOZ vs. non-SOZ | Mann–Whitney U test | 0.970(Cohen’s d) | 102 vs. 784 | 0.05 | 1 |
|  | peri-SOZ vs. non-SOZ | Mann–Whitney U test | 0.467(Cohen’s d) | 249 vs. 784 | 0.05 | 0.999 |
|  | SOZ localization (SOZ vs. peri-&non-SOZ) | Binominal logistic regression analysis | 0.441(Cohen’s d) | 102 vs. 1033 | 0.05 | 1 |
|  | SOZ localization (SOZ vs. peri-SOZ) | Binominal logistic regression analysis | 0.270(Cohen’s d) | 102 vs. 249 | 0.05 | 0.946 |
| Abbreviations: MI, modulation index; SOZ, seizure onset zone. | |  |  |  |  |  |

| **Table S3. Post hoc power analysis for comparion of MI_FRs/slow waves_ across SOZ, peri-SOZ, non-SOZ.** | | |  |  |  |  |  |
| --- | --- | --- | --- | --- | --- | --- | --- |
| MI_HFOs/slow waves_ | Comparison | Statistical test | Effect size (Cohen’s d / η²) | Sample size (n/group) | α level | Power (1–β) |  |
|  |  |  |  |  |  |  |  |
| MI_FRs/1-2 Hz_ | SOZ vs. peri-SOZ vs non-SOZ | Kruskal–Wallis test | 0.033(η^2^) | 102 vs. 249 vs. 784 | 0.05 | 0.999 |  |
|  | SOZ vs. peri-SOZ | Mann–Whitney U test | 0.597(Cohen’s d) | 102 vs. 249 | 0.05 | 0.989 |  |
|  | SOZ vs. non-SOZ | Mann–Whitney U test | 0.497(Cohen’s d) | 102 vs. 784 | 0.05 | 0.942 |  |
|  | peri-SOZ vs. non-SOZ | Mann–Whitney U test | -0.123(Cohen’s d) | 249 vs. 784 | 0.05 | 0.279 |  |
|  | SOZ localization (SOZ vs. peri-&non-SOZ) | Binominal logistic regression analysis | 2.196(Cohen’s d) | 102 vs. 1033 | 0.05 | 1 |  |
|  | SOZ localization (SOZ vs. peri-SOZ) | Binominal logistic regression analysis | 2.704Cohen’s d) | 102 vs. 249 | 0.05 | 1 |  |
| MI_FRs/2-3 Hz_ | SOZ vs. peri-SOZ vs non-SOZ | Kruskal–Wallis test | 0.040(η^2^) | 102 vs. 249 vs. 784 | 0.05 | 1 |  |
|  | SOZ vs. peri-SOZ | Mann–Whitney U test | 0.642(Cohen’s d) | 102 vs. 249 | 0.05 | 0.995 |  |
|  | SOZ vs. non-SOZ | Mann–Whitney U test | 0.643(Cohen’s d) | 102 vs. 784 | 0.05 | 0.995 |  |
|  | peri-SOZ vs. non-SOZ | Mann–Whitney U test | -0.040(Cohen’s d) | 249 vs. 784 | 0.05 | 0.073 |  |
|  | SOZ localization (SOZ vs. peri-&non-SOZ) | Binominal logistic regression analysis | 4.181(Cohen’s d) | 102 vs. 1033 | 0.05 | 1 |  |
|  | SOZ localization (SOZ vs. peri-SOZ) | Binominal logistic regression analysis | 4.163(Cohen’s d) | 102 vs. 249 | 0.05 | 1 |  |
| MI_FRs/3-4 Hz_ | SOZ vs. peri-SOZ vs non-SOZ | Kruskal–Wallis test | 0.066(η^2^) | 102 vs. 249 vs. 784 | 0.05 | 1 |  |
|  | SOZ vs. peri-SOZ | Mann–Whitney U test | 0.850(Cohen’s d) | 102 vs. 249 | 0.05 | 1 |  |
|  | SOZ vs. non-SOZ | Mann–Whitney U test | 0.814(Cohen’s d) | 102 vs. 784 | 0.05 | 1 |  |
|  | peri-SOZ vs. non-SOZ | Mann–Whitney U test | -0.110(Cohen’s d) | 249 vs. 784 | 0.05 | 0.23 |  |
|  | SOZ localization (SOZ vs. peri-&non-SOZ) | Binominal logistic regression analysis | 7.860(Cohen’s d) | 102 vs. 1033 | 0.05 | 1 |  |
|  | SOZ localization (SOZ vs. peri-SOZ) | Binominal logistic regression analysis | 8.147(Cohen’s d) | 102 vs. 249 | 0.05 | 1 |  |
| MI_FRs/4-8 Hz_ | SOZ vs. peri-SOZ vs non-SOZ | Kruskal–Wallis test | 0.097(η^2^) | 102 vs. 249 vs. 784 | 0.05 | 1 |  |
|  | SOZ vs. peri-SOZ | Mann–Whitney U test | 0.713(Cohen’s d) | 102 vs. 249 | 0.05 | 1 |  |
|  | SOZ vs. non-SOZ | Mann–Whitney U test | 0.780(Cohen’s d) | 102 vs. 784 | 0.05 | 1 |  |
|  | peri-SOZ vs. non-SOZ | Mann–Whitney U test | 0.960(Cohen’s d) | 249 vs. 784 | 0.05 | 0.449 |  |
|  | SOZ localization (SOZ vs. peri-&non-SOZ) | Binominal logistic regression analysis | 3.681(Cohen’s d) | 102 vs. 1033 | 0.05 | 1 |  |
|  | SOZ localization (SOZ vs. peri-SOZ) | Binominal logistic regression analysis | 3.119(Cohen’s d) | 102 vs. 249 | 0.05 | 1 |  |
| Abbreviations: FRs, fast ripples; MI, modulation index; SOZ, seizure onset zone. | | |  |  |  |  |  |
